# Enhancing Rheological and Textural Properties of Gelatin-Based Composite Gels through Incorporation of Sesame Seed Oleosome-Protein Fillers

**DOI:** 10.3390/gels9100774

**Published:** 2023-09-23

**Authors:** Fatemeh Sheikh, Maryam Hasani, Hossein Kiani, Mohammad Javad Asadollahzadeh, Farzaneh Sabbagh

**Affiliations:** 1 Department of Food Science and Technology, Shahrood Branch, Islamic Azad University, Shahrood 3619943189, Iran; sheikhf2006@yahoo.com; 2 Bioprocessing and Biodetection Lab (BBL), Department of Food Science and Technology, College of Agriculture and Natural Resources, University of Tehran, Karaj 31587-77871, Iran; hokiani@ut.ac.ir; 3 Department of Chemical Engineering, Shahrood Branch, Islamic Azad University, Shahrood 3619943189, Iran; m.asadalahzadeh@gmail.com; 4 Department of Botany and Plant Science, Faculty of Biological Science, Alzahra University, Tehran 1993891176, Iran

**Keywords:** sesame, oleosome, composite gel, viscoelastic properties

## Abstract

In this study, the protein and oleosomes of sesame seeds were extracted individually and used to prepare a gel composed of gelatin, protein, and oleosomes. Mixtures of gelatin and sesame seeds protein were prepared, and oleosomes with different percentages (0, 10, 20 and 30% of their weight) were used. Different amounts of oleosomes in the composite gel samples were examined for their morphological, rheological, and textural properties. The results of the viscoelastic properties of different composite gel samples indicated that a higher percentage of oleosomes would increase the storage modulus (G′), loss modulus (G″), and complex viscosity (η*). The storage modulus of all gel samples was greater than the loss modulus, suggesting a solid behavior. So, in the sample with 30% oleosome, the storage modulus and the loss modulus reached 143,440 Pascals and 44,530 Pascals. The hardness and breaking force in samples containing 30% oleosome reached 1.29 ± 0.02 and 0.17 ± 0.02, respectively. In general, it can be said that composite gels based on gelatin-sesame seed protein modified with oleosome can be used as a part of food components in various dairy products, gelatin desserts, lean meat products and the production of useful products.

## 1. Introduction

Oleosomes consist of a core of triglycerides surrounded by a membrane of protein-phospholipid mixture with distinguished proteins (e.g., oleosins, caleosins, and steroleosins). They have a spherical shape with an average diameter in the range of 0.2–2.5 μm. For example, soybean oleosomes have a composition of 91.92% lipid, 7.85% protein, and 0.80% phospholipid based on dry weight [1]. Moreover, oleosomes may contain some minor bioactive components like phytosterols, tocopherols, essential amino acids, isoflavones, and minor proteins such as dioxygenase and lipoxygenase [2].

Besides carrying hydrophobic molecules, the oily network of oleosomes can also carry antioxidants, steroids, hormones, and cosmetic lipids. Oleosomes stability influences their application in numerous fields. Improved stability of oleosomes lowers the cost of commercial and industrial processes since the decomposition of oil bodies is reduced [3]. Ultrafiltration or centrifugation can be used to improve the oil droplet content of the extract or to recover the oil droplets as a thick cream. Oleosome-containing emulsions (condensed cream) are useful as components in various products such as food [4] or as a component in food products. Furthermore, since aqueous extracted oleosomes have high physicochemical stability, oxidation-sensitive nutrients such as phenols, vitamins, and unsaturated fatty acids are well stored in them [5]. The gels contained in food products may consist of biopolymer networks containing fat particles. In the gel, fat particles act as brittle structural agents. Thus, to assess the durability of the rheological properties of composite gels for both strength and refractive properties, type and amount of fat. The gel rheology depends on particle size and particle-gel matrix interactions. Fat particles act as inactive fillers and reduce the mechanical properties of the gel when there are no interactions [4]. The fat particles, however, act as active fillers when they interact with a network of surrounding molecules, giving the gel greater mechanical properties, and causing it to harden. Based on the concept of bonded and non-bonded particles, it is possible to distinguish between active and inactive filler droplets [6]. Given the presence or absence of interaction between the gel matrix and emulsion droplet, filler particles may alter the structure or properties of the gel. With increasing the fraction of the particles with a high-volume fraction, the degree of particle clustering may also enhance the gel strength [7]. Oily compounds are commonly extracted using organic solvents and high temperatures. Environmental pollution and a significant decline in the quality of extracted products are associated with these processes [8,9]. The noteworthy qualities of the oleosomes from natural sustainable resources, including high emulsifying and interfacial activities, low toxicity, biocompatibility, cost-competitive advantage and high oxidative stability have attracted increasing attention to use them to substitute synthetic emulsifiers to prepare food emulsions. The use of oleosomes as a substitute for oil compounds in food products has been considered to fulfill consumer demand for “all-natural” products. For example, the inclusion of plant-derived oleosomes in the production of dairy products, mayonnaise, chocolate, and soy milk showed excellent stability with high nutritional value [10,11]. From an industrial point of view, the use of oleosomes as an oil substitute in various products has grown significantly, and the use of oleosomes extracted from plants in the chocolate and soy milk industry has increased stability and improved nutritional value. Kirimlidou et al. showed that sesame and hazelnut oleosomes are excellent substitutes for common fat droplets and particles and can be well used in the protein-gelatin gel network [4]. In recent years, there has been a growing interest in the development of innovative green refinery processes to prepare oleosomes from oilseeds of nutritional interests (sesame seed, linseed, rapeseed and sunflower) for food applications. The processes including the soaking of the seeds, grinding and centrifugation have been successfully used to extract oleosomes along with proteins by aqueous extraction from oilseeds [12]. Yang et al. investigated the rheological properties of the prepared emulsion gel with soybean oleosomes as oil droplet filler and carrageenan as gel matrix, they found that emulsion gels containing oleosome show spring lubrication properties. These researchers observed that the amount of oleosome and the pH of the environment are effective on the formation temperature and gel strength [13]. This study examines the effect of sesame oil oleosomes on the formation of a composite gel network and its rheological properties. In this study, sesame protein and gelatin will also serve as the building blocks of protein structure in the composite gel network. Thus, the present study aims to preparation of composite gels using sesame protein and gelatin and analyze of rheological, textural and microstructural properties of composite gels.

## 2. Results and Discussion

### 2.1. Chemical Properties of Sesame Seeds

Based on the results, the moisture, ash, fat, protein, and carbohydrate contents of sesame seeds were 5 ± 0.01, 4.55 ± 0.71, 53 ± 0.02, 19.59 ± 1.34, and 13.23 ± 0.12% respectively. In addition, the protein extraction efficiency of sesame seeds was 21 ± 1.23%.

### 2.2. Physicochemical Properties of Oleosomes Extracted from Sesame Seeds

**Figure 1:**
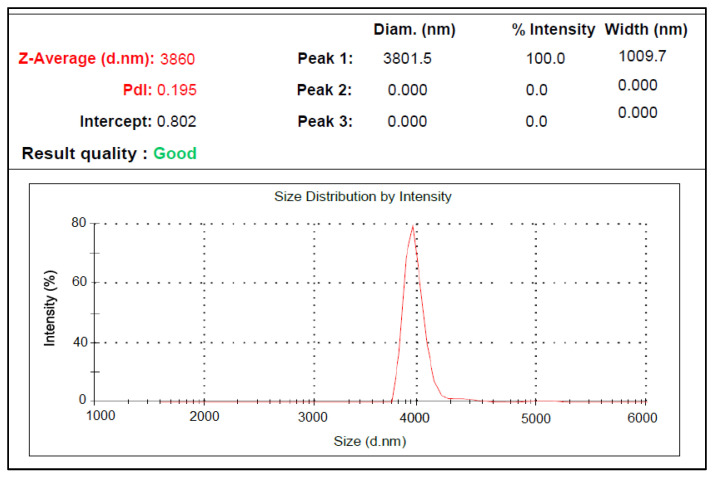
Sesame seed oleosomes PDI index and particle size distribution.

The results of the analysis revealed that the moisture, fat, and protein contents of oleosomes extracted from sesame seeds were 25.64 ± 0.05, 73.98 ± 0.08, and 0.71 ± 0.04%, respectively. In addition, the yield of oleosome extraction from sesame seeds was equal to 68.96 ± 1.41%. The polydispersity index (PDI) and particle size distribution results of oleosome samples from sesame seeds are presented in Figure 1.

**Figure 2:**
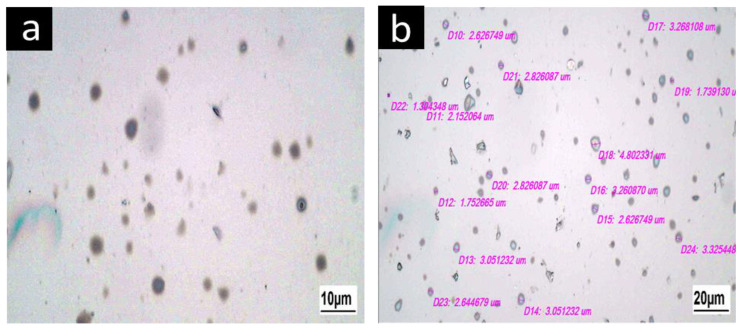
Optical microscopy images of sesame seeds oleosomes, at 60× (**b**) and 100× (**a**) magnifications.

Figure 1 shows that the mean particle size of oleosomes extracted from sesame seeds was 3.86 μm and the dispersion index was 0.195. The particle size of the oleosome was 5 μm, which is in agreement with the results of Kirimlidou et al. [4]. According to Kirimlidou et al. oleosomes can appear large due to clustering and cohesiveness, but they are frequently smaller than 10 μm in size [4]. Figure 2 reveals the images of oleosomes extracted from sesame seeds and their approximate size. Oleosomes from sesame seeds were 3.8 μm, as shown in Figure 2. DLS results were confirmed by optical microscopy.

### 2.3. Sesame Oleosome Proteins and Their Properties

**Figure 3:**
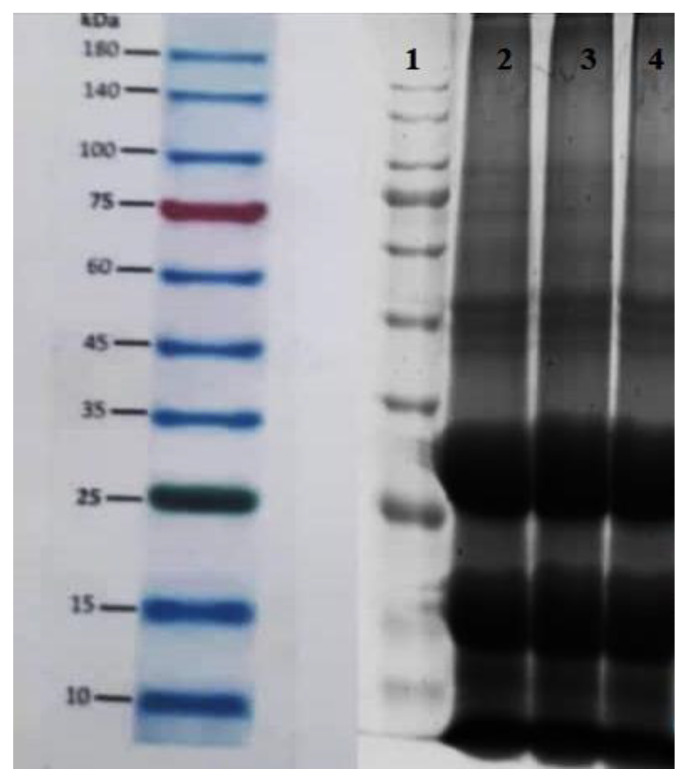
Electrophoresis of sesame seed oleosome protein.

To determine the structure of sesame oleosome protein, electrophoresis was used. Protein compounds were detected by a coma-blue color as displayed in Figure 3 in the second to fourth columns, Three replicates of the sesame protein sample have been shown with three thick bands. The first thick band in these columns is shown within 8 to 10 kDa, the second thick band within 15 to 20 kDa, and the next thick within 25 to 35 kDa. The protein was measured between 20 and 26 kDa, which relates to oleosins and caleosins. According to Kirimlidou et al. the rest of the bands are related to albumin and other globulins [4]. Oleosins, caleosins, and steroleosins are the main proteins found in the oleosomal membrane. Oleosin is a hydrophobic protein with a low molecular weight of 15–26 kDa. Oleosins play an important role in regulating the oleosome size. The oleosome contains 12% oleosins, while the total protein content is 20 to 25%. Oleosins have significant surface activity as they have a long central portion that is hydrophobic, helping them penetrate triglyceride nuclei, and since their N- and C-terminal residues facilitate contact with phospholipid polar heads. Oleosin plays an essential role in the stabilization of oleosomes by interacting with oleosome polar phospholipids and repelling them electrostatically. The molecular weight of calosin is 25–35 kDa. Compared with oleosin, this protein has a shorter hydrophobic region and a longer hydrophilic region. The N-terminal portion of caleosine is hydrophilic and contains a Ca^+2^ binding site that is exposed to a continuous aqueous phase.

Steroleosins are relatively large proteins that attach to the surface membrane of oleosomes with a molecular weight of greater than 35 kDa. This protein has a hydrophobic fraction equal to the length of caleosin and a long hydrophilic fraction that is inclined towards the cytosol. Two isoforms of steroleosin, steroleosin A and steroleosin B have been identified in sesame oleosomes, and steroleosin B in peanut oleosomes with molecular weight 40 kDa, which are responsible for oleosome formation and degradation. Oleosome membrane phospholipids are also considered important for surface stability [2,14]. The molecular weights of soybean and peanut oleosome proteins are mostly between 15–30 and 35–50, 15–25, and 30–37 kDa, while those of sunflower protein are 15–25, 35–45 and 50–75, respectively. The bands below 15 kDa were attributed to the napin-type albumins that contain two basic subunits, i.e., a long-chain subunit (about 10 kDa) and a short-chain subunit (about 4.5 kDa). This is in agreement with studies on the protein [12]. The oleosome not only contains the structural proteins on the surface of the oleosome, but also carries various external proteins, which could be due to differences in surface protein compositions [15]. 

### 2.4. Zeta Potential of Sesame Seed Oleosomes

**Figure 4:**
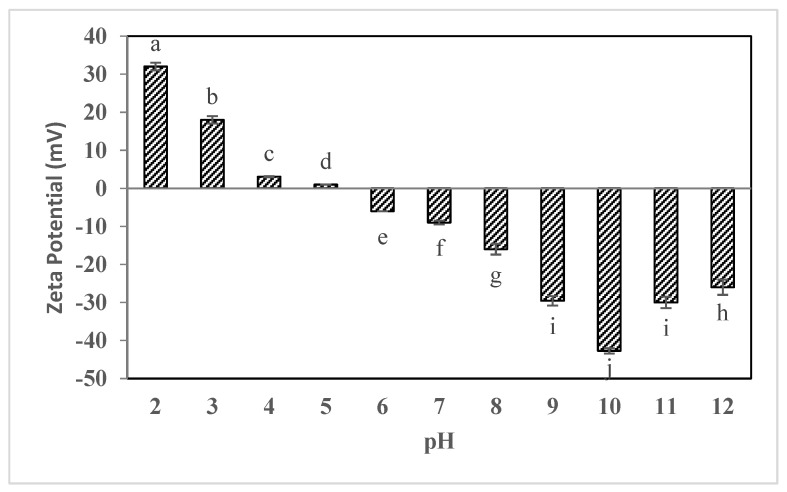
Zeta potential changes of oleosome treatments extracted from sesame seeds in pH range 2 to 12. Different lowercase letters indicate a significant difference (*p* < 0.05) at the 95% probability level in each treatment.

Figure 4 displays the zeta potential changes of sesame seed oleosomes when pH has changed from 2 to 12. Based on the results, it was found that changes in the zeta potential of sesame seed oleosome samples have been significantly (*p* < 0.05) dependent on the pH of the environment. Thus, as shown in Figure 4, at acidic pHs (below 5), the oleosome samples extracted from sesame seeds had a positive zeta potential, and as the pH increased towards alkaline pHs, the zeta potential changed to negative. The zeta potential of the oleosome was positive when the pH was below 5, and reduced to 0 mV between pH 5–6, which reached the isoelectric point of the oil bodies. Thus, as shown in Figure 4, the lowest magnitude of the zeta potential at pH was 5 (0.98 ± 0.02 mV) and the maximum magnitude of the zeta potential (−42.17 ± 1.23 mV) for oleosome samples of sesame seeds was observed at pH = 10. The zeta potential is one of the factors that affect the stability of samples in emulsions and suspensions. Studies have reported that increased zeta potential results in more stable emulsions and colloids. Zeta potential magnitude grows when a particle accumulates more charge on its surface, which results in electrostatic repulsion. Due to the high electrostatic repulsion force at zeta potentials less than −30 mV as well as above +30 mV, the tendency to particle accumulation is greatly reduced. Under acidic conditions (pH less than 4), the protein portion of the oleosomes extracted from sesame seeds is protonated, which will be accompanied by a positive charge in the oleosome samples. On the other hand, oleosomes undergo protonation, which results in negative charges being formed on their surface and a negative zeta potential [16]. At pHs 4 and 5, especially at pH 5, the lowest amount of zeta potential, or in other words, the lowest surface charge was observed in oleosome samples, so isoelectric pH is probably obtained for these samples. 

Other researchers have also found similar results. Sukhotu et al. found that the isoelectric point (IEP) of peanut oil bodies is located between pH 4 to 5. The zeta potential of oil bodies that are related to the net electric charge of protein on the oil body surface depends on pH. And the net electric charge changed from positive to negative values with increasing pH [17]. Many researchers reported when the pH values of oil body suspension are far away from the isoelectric point, electrostatic repulsion should be much greater than that at IEP [17,18,19]. A previous study on IEP of oil bodies and zeta potential from maize germ, soybeans and Oryza sativa oil bodies where IEP was found at a pH between 3 and 5 [20,21,22,23]. Similar to the results recorded by Tzen et al. have found that the isoelectric points of the oil body from different plant sources are approximately pH 5–6. The difference in isoelectric point can be related to the difference in extraction method and seed sources [24].

### 2.5. Creaming Index of Sesame Seed Oleosomes

**Figure 5:**
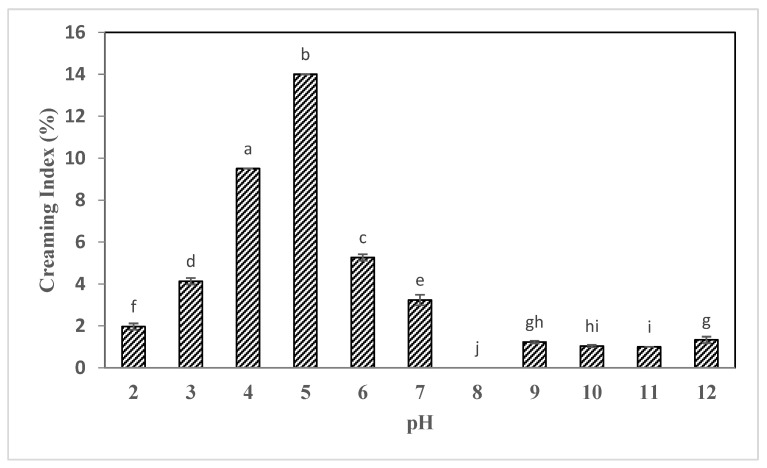
Changes in creaming index in different pH ranges of sesame seed oleosomes. Different lowercase letters indicate a significant difference (*p* < 0.05) at the 95% probability level in each treatment.

According to the results, it was found that changes in the creaming index were significantly (*p* < 0.05) affected by the pH of the medium. As displayed in Figure 5, the creaming index in samples increased as pH rose from 2 to 5 (acidic). The maximum percentage of creaming (14%) was associated with the pH = 5 suspension and the lowest percentage (without any separation of serum) with the samples stored at pH = 8. The highest creaming index (14.37 ± 1.25%) was observed for the suspensions stored at pH = 5 and the lowest creaming rate for the samples stored at pH = 2, 10, and 11. This test confirms the results reported in the section on zeta potential. Zeta potential is one of the factors influencing suspension stability, as mentioned previously. Increasing zeta potential results in the accumulation of large like charges on the surfaces of the particles, resulting in a strong electrostatic repulsion force between particles, enhancing the stability and reducing two-phase samples. The maximum value of zeta potential was found at pHs between 10 and 11, which had also the lowest creaming index. According to these results, the changes in creaming index and zeta potential were in agreement with other researchers’ findings. Qi et al. found that soybean oleosomes suspension stored at alkaline pHs and acidic pHs had the highest stability compared to samples stored at pHs 3 to 5 [25]. Previous studies showed that for low pH values the proteins corresponding mainly to oleosins located at the surface of the oleosomes were involved in the formation of the aggregates. Coalescence of oleosomes was also observed at low pH values, indicating fusion of natural oleosomes to form large oleosomes. Decreasing the pH (increasing the amount of H^+^ cations) altered the physical stability of the natural oil-in-water emulsions by inducing aggregation of oleosomes as a result of the decrease in the electrostatic repulsions between oleosomes associated with low negative potential values. The formation of large aggregates of oleosomes favours their phase separation from the aqueous phase and as a result, they rose to the top of the containers (due to the low density of lipid-rich oleosomes). These results showed the impact of pH on the physical stability of chia seed and camelina seed oleosomes, with the formation of aggregates of oleosomes near the IEP, and are in agreement with the literature on other sources of oleosomes [12].

### 2.6. Analysis of the Properties of Composite Gels

#### 2.6.1. Rheological Properties of Composite Gels

**Figure 6:**
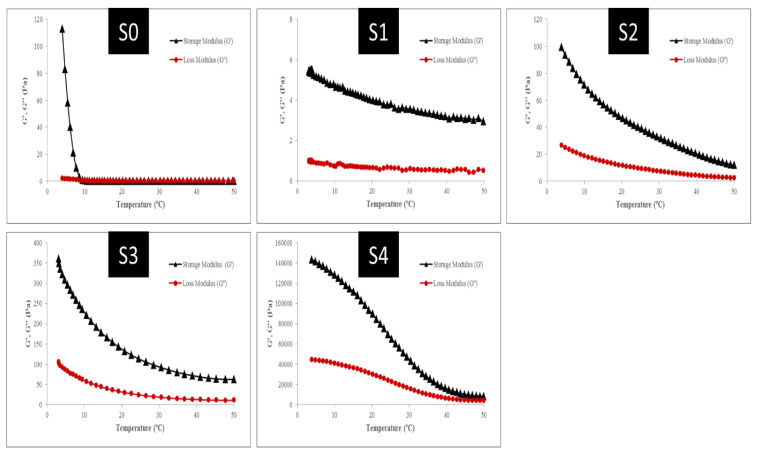
Changes in the dynamic modulus (G′ and G′′) of mixed gelatin, sesame seed protein, and sesame seed oleosomes based on temperature changes (temperature reduction from 50 to 4 °C).

From a technological, engineering, and sensory perspective, the rheological properties of food are very important. These properties influence the sensory characteristics of consumers in addition to guiding the development of food processes and formulations. Thus, understanding the rheological behavior of food products is crucial in this field [26]. Rheological characteristics of the composite gel, such as storage modulus (G′), loss modulus (G″), and complex viscosity (η*) were shown in Figure 6. When the values of G″ < G′ (gel properties), the elastic behavior dominates the viscous behavior, and when the values of G″ > G′ (liquid behavior), the viscous behavior dominates the elastic behavior. In the cooling process, the intersection points of G′ and G″ (G′ = G″) is defined as the point of gel formation. During the cooling process, when the gel formation has not yet occurred, the G′ value is lower than the G″ value. Then, G′ increases at a certain temperature and exceeds G″ which indicates the gel formation [13]. According to the results obtained in the present study, it was observed that G′ and G″ did not intersect at any point in the temperature range (50 to 4 °C) and by adding oleosome the difference between the values of G″ and G′ have increased at the temperature of 50 °C. Therefore, it can be seen that with the use of oleosome in the gel structure, the gelation point temperature increases. In the control (S0), at a temperature of 50 °C, the loss and storage modulus were interrupted, which can be considered as the gel formation point for pure gelatin [27,28]. It was determined that in the S1 treatment, in which no oleosomes were used in the gel preparation, the storage modulus of this sample was higher than the loss modulus indicating a solid-like behavior within the temperature range. The storage modulus increased when the temperature decreased. Also, the elastic behavior of this sample was superior to its viscose behavior, and there were no points of intersection between them. The storage modulus changed with temperature elevation, though the changes were small and reached 5.45 Pascals at about 4 °C. The storage modulus and loss modulus increased with the reduction of temperature as well as when oleosomes were added from 10 to 20% for S2 and S3, respectively. In the S2, the value of storage modulus showed a slight increase at about 25 °C, and the storage and loss modulus were reported at 4 °C (99.3 and 26.5 Pascals, respectively). In the S3, increasing the amount of oleosome to 20% caused an increase in the storage and loss modulus at around 4 °C (361 and 106 Pascals, respectively). At 4 °C, the storage and loss modulus were 143,440 and 44,530 Pascals in the S4 with 30% oleosome content. It was found that the presence of 30% oleosome significantly affected storage and loss modules. Oleosomes, at this concentration, could be distributed well in the system and have an obvious effect on elastic response strength. The sample also solidified at higher temperatures with this concentration of oleosomes. The presence of gelatin, protein, and sesame seed oleosome strengthens the structure of the gel network. As oleosome disperses in this network, due to its emulsifying and hydrophobic properties, oleosome particles are placed in the form of a bridge between polymer chains and water molecules and provide a suitable three-dimensional network for trapping water molecules. By increasing the amount of oleosome up to 30% in treatments with the same amount of sesame protein and gelatin, due to the use of less water, the concentration of protein in the continuous aqueous phase increases and improves the strength of the gel. By reducing the temperature of gelatin solutions, the expansion of the triple helix connection areas stimulates network formation. The oleosomes placed in the gel structure have a positive effect on the gelation time and temperature and increase the network formation. Probably, the presence of oleosomes leads to a decrease in protein mobility and provides the possibility of faster formation of connection areas. Therefore, oleosomes act as strong and active filament fillers that enhance the strength of the composite gel structure [4].

**Figure 7:**
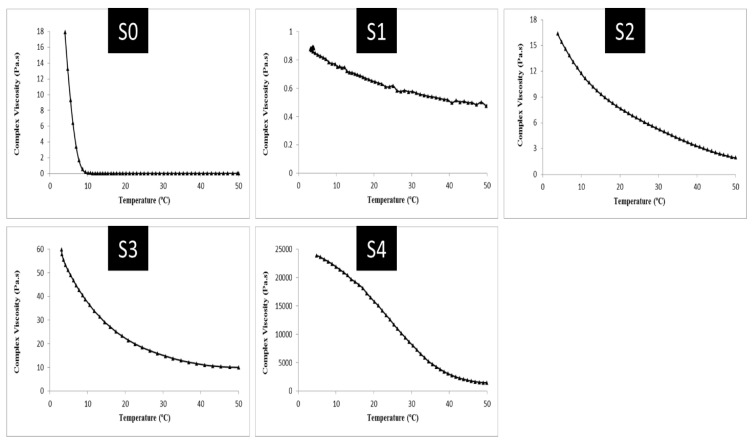
Changes in viscosity of complex gelatin gels, sesame seed protein, and sesame seed oleosomes with decreasing temperature (from 50 to 4 °C).

Figure 7 indicates the changes in the viscosity of the composite gel complexes. Thus, as shown in Figure 7, the changes in the viscosity of the complex gel samples would follow a similar trend to the variations in the storage modulus and loss modulus indices. The viscosity of the complex increased from 50 to 4 °C in all samples. In S0 (pure gelatin), the viscosity grew significantly from about 7 °C to 4 °C and reached 17.9 Pa.s. This amount was reported to be 0.88 Pa.s at 4 °C in the S1, where there were equal amounts of gelatin and sesame protein. As the amount of oleosome in the S2, S3, and S4 samples increased from 10 to 30%, the viscosity of the complex was reported as 16.35, 59.8, and 23,904 Pascal seconds, respectively. The storage modulus was also found to be higher in the composite gel samples and control gel sample (S0) than the loss modulus. Additionally, the use of oleosomes in the formulation of composite gels significantly affected the storage and loss modulus. Within the studied temperature range, no crossover occurred between loss and storage modules. As shown for the composite gels, the storage modulus (G′) was greater than the loss modulus (G″), suggesting that the control gels and composite gels had an interconnected structure (gels) of mainly elastic nature. The higher storage modulus confirms the solid behavior of these gels, which store them in response to force application and do not undergo much deformation. 

Sesame protein is soluble and has a high molecular weight, which allows it to retain more water, increasing protein-protein interactions [29]. Mixing sesame protein and gelatin will increase the hydrodynamic volume occupied by these biopolymers. By increasing the amount of protein in a solution, the hydrodynamic volume will elevate the concentration of another polymer in the remaining solution [28,30]. Pang et al. found similar results. It was found that gelatin-based gel formulations with milk proteins had a higher storage modulus. Scientists suggest that milk proteins could create and alter hydrogen bonds in the gelatin network, strengthening the gel network [31]. The higher G′ and G″ indices in response to the use of protein and sesame seed oleosome suggested that oleosome had been well dispersed in the gel network structure. Oleosomes act as a bridge between polymer chains and water molecules by emulsifying and hydrophobic properties, and they form a three-dimensional structure that traps water molecules. Viscoelastic properties are also affected by complex viscosity (η*). It is an estimate of viscoelastic behavior (gel strength) based on the slope of the complexity viscosity diagram. It is influenced by several factors such as the concentration of hydrocolloids used as stabilizers. In other words, the higher the slope on the complex viscosity graph, the greater and better the gel strength [32]. As displayed in Figure 7 changes in the complex viscosity of the composite gel samples also revealed that variations in viscosity had a similar trend to changes in storage modulus and loss modulus indices. Thus, the results revealed that increasing the number of oleosomes in the formulation of composite gels up to 30% led to an increase in the viscosity of their complex. Zhang and Zhao examined the effects of incorporating soy protein isolate into the structure of gels based on gelatin. In the study, they indicated that soy protein in the structure of composite gels increased G-indices and apparent viscosity. They also found that the use of transglutaminase enzymes and crosslinking between polymer chains caused enhanced gel structure and viscoelastic parameters. They attributed it to the hydrodynamic volume occupied by the protein polymers, which augments the viscosity and strengthens the structure [33].

#### 2.6.2. Gel Texture

Changes in texture parameters such as hardness, adhesiveness, and breaking force in composite gel formulations are shown in Table 1. It was found that the hardness of treatment S1 was 0.22 ± 0.01 N and it also significantly (*p* < 0.05) increased with increasing the amount of oleosomes in the samples. Adhesiveness values had a decreasing trend with increasing hardness in the S2, S3, and S4 samples. The lowest hardness in composite gel samples was equaled 0.22 ± 0.01 N and the highest was equaled to 1.29 ± 0.02 N, which were related to S1 and S4 treatments, respectively. The breaking force in the gels also increased by increasing the amount of oleosomes in the structure of the gels. The lowest amount of breaking force was observed in the S0 and S1 treatments, while there was no statistically significant difference between the S0 to S3 treatments (*p* < 0.05). The hardness of the gel is known as the maximum height of the plunger penetration curve. Adhesive force is the maximum negative force required to get the plunger out of the gel, and the breaking force is defined as the first significant rupture in the plunger diagram inside the gel at a total displacement of 15 mm. Increasing the amount of oleosome led to increased tissue hardness and stronger gel. Therefore, it can be said that with increasing hardness, the resistance to failure also increases, which will lead to less deformation of the gel texture. Increasing the utilization of sesame seed oleosome increased the rheological indices G′, G″ and the complex viscosity of the composite gel samples. The same was true for the mechanical indices of the tissue. Proteins and oleosomes increased the hardness of the texture and the oleosomes, due to their emulsifying properties. They also strengthen the structure of the gel, as these compounds act as a bridge between the components of the gel, which is associated with the hardness of the texture. The oily nature of oleosomes causes these compounds to act as lubricants when used at concentrations higher than the optimum concentration, reducing the hardness of the gel tissue [28,34,35]. The results obtained during this study were consistent with the findings of other researchers in terms of texture properties. In agreement with previous studies, the addition of the oleosomes significantly affected the structural parameters of the emulsion-filled gels. Liao et al. found that when the concentrations of the oleosomes reached 35%, the hardness of the gel formed was the highest, compared to other concentrations [36]. This was because the density of the physical bonds, which enhanced the emulsion-filled gel network to avoid damage, increased with the oleosomes content of the gel [37]. 

#### 2.6.3. Microstructural Properties 

**Figure 8:**
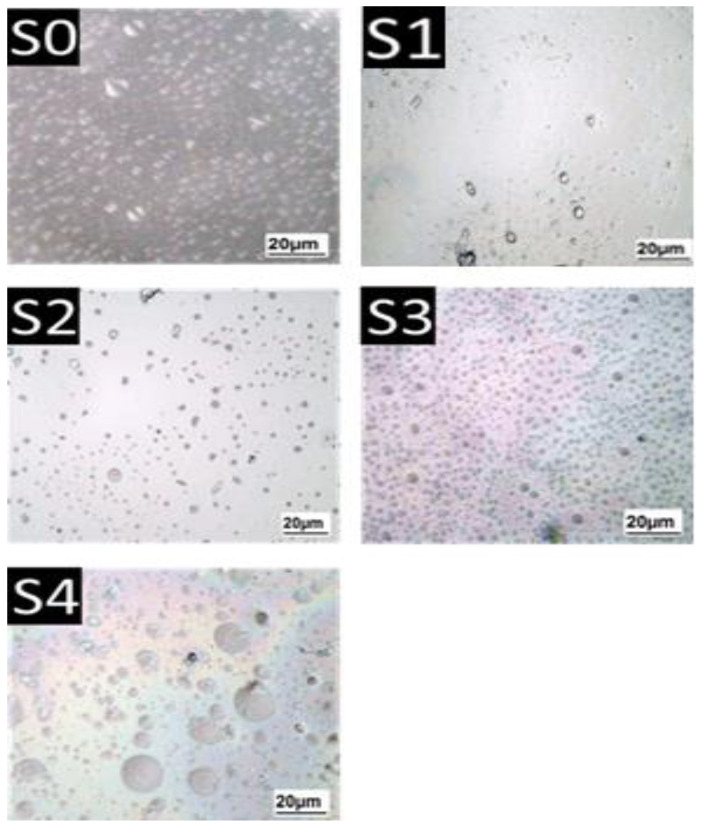
Microstructure of a mixture of gelatin, sesame seed protein and sesame seed oleosome for samples.

Under a light microscope, S0 (pure gelatin) and gel samples containing sesame seed protein and oleosome were analyzed. Figure 8 depicts that in the S0, the droplets were distributed evenly throughout the gelatin structure. Pure gelatin had a very loose structure and was easily broken. In S1, it was observed that the droplets were finer in the network and probably a completely uniform texture was not observed due to the absence of oleosomes. In S2, S3, and S4, the oleosomes were evenly distributed within the gel structure. Further, in S4, where the amount of oleosomes was higher, the size of oleosomes also increased, as seen in part of the accumulation shape. The oleosomes were relatively uniformly distributed in the gelatinous matrix and sesame protein, as displayed in Figure 8. Although light microscopy cannot be used to estimate the size of oleosomes, its images show that they have been similar to the original emulsion regarding size. During the emulsification process, no extensive accumulation took place, and the particle size distribution improved with an increase in the sesame oleosome content, suggesting that the emulsifying properties of sesame oleosome have improved. A combination of oleosomes and sesame protein resulted in a uniform distribution of emulsion molecules due to the surface proteins on the oleosome. These surface proteins would enhance emulsion stability and improve the physicochemical properties of the gel [4,28]. In the S0, no scattered droplets or particle aggregation were observed. The chains of gelatin contain proline and hydroxyproline, where the bonds between the chains are hydrogen [30]. As in S1, a relatively uniform structure was created from the combination of gelatin and sesame protein, which was not observed probably due to the lack of a completely uniform texture oleosome. Generally, sesame protein accumulates and forms a dense state, but in combination with gelatin, the accumulation of proteins is reduced. Pang et al. found that gelatin concentrations, pH, and the addition of milk proteins would affect gelation behavior, microscopic structure, texture, and rheology. At low concentrations of gelatin, it showed a soft and brittle texture, and upon adding 2% of milk protein compounds, the gel found good resistance to brittleness; the higher the gelatin concentration, the stronger the texture in the gel [31].

In S2 to S4, the structure of the gel has improved by increasing the amount of oleosome in the gel structure, and by increasing the amount of oleosome to 20%, the number of oleosomes per unit area has increased. So that the oleosomes are dispersed individually and relatively uniformly in the gelatin matrix. While in the gel structure with 30% oleosome, the number of oleosomes decreased per unit area and their size increased. According to Nikiforidis et al. sesame oleosomes provide a better structure and texture to gels due to their phospholipid compounds and active surface proteins. Oleosomes are generally resistant to aggregation, coagulation, as well as adhesion, and are relatively resistant to environmental pressures [11]. By contrast, the presence of gelatin stimulates depletion flocculation in the oleosome-gelatin matrix. The phenomenon of discharge in gelatin-containing solutions is well known. Evacuation interactions will create a tendency for moving oily objects to move toward each other. A second explanation for the heterogeneous structure of composite gels could be the improper mixing of gelatin, sesame protein, and oleosome at the beginning of the process. Because of the relatively high-volume fraction of oil droplets in the mixture, improper mixing could lead to orthosynthetic accumulation of oil droplets, as observed in S4. If the surface composition of oleosome droplets is bound to a continuous protein matrix, oleosome droplet fusion has a different effect on the final rheological properties of gels. The active filler effect and hardness of the composite gel are enhanced when the gelatin matrix is bonded to the oleosome droplets. In general, the gel structure is expected to be strengthened when the droplets accumulate due to the effective expansion in oil volume. Kirimlidou et al. reported that the binding of oleosome droplets could change the viscoelastic properties of gelatin composite gels. The increase in oleosome by weight/volume means that storage and loss modulus have increased. Oleosomes act as fillers and improve the strength of composite gels, while the surface proteins of oleosomes interact with the main matrix, strengthening it [4,28]. This study indicated that the storage and loss modulus of samples with oleosome percentage increased significantly, confirming the results of Nikiforidis et al. as well as Kirimlidou et al. [4,11].

## 3. Conclusions

Sesame contains a wide range of nutritional and bioactive substances with remarkable functional properties. Protein and oleosomal compounds in this valuable seed make it suitable for use in food formulations. A gelatin-based composite gel formulation was developed by using sesame seed protein and sesame seed oleosomes first extracted from sesame seeds. Based on evaluating the viscoelastic properties of composite gels, it was found that the use of sesame seed oleosome in the formulation of composite gels increased their G′, G″, and complex viscosities. Furthermore, the temperature reduction from 50 to 4 °C caused elevation of G′, G″, and the viscosity of the complex. The results presented that by increasing the amount of oleosome in the gel structure, the gel hardness and breaking force increased and the adhesiveness decreased significantly. The microstructure studies of the prepared gels showed that in the gels containing oleosome, the structure became denser and the oleosomes were uniformly dispersed in the gel structure. Due to the presence of surface-active proteins and phospholipid compounds, oleosomes can improve the structure and texture of the gel. In general, it can be suggested that composite gels based on gelatin-sesame seed protein modified with oleosome can be used as a food component in dairy products, desserts, and jellied meat products as a functional food.

## 4. Materials and Methods

### 4.1. Materials and Equipment

Sesame seeds were obtained from the local market (Tehran, Iran). All chemicals were provided by Merck Company (Darmstadt, Germany). Cow gelatin powder (250 bloom, alkaline), centrifuge and mill (IKA, Staufen, Germany), pH meter (Farnell, West Yorkshire, England), oven (Memert, Bavaria, Germany), Anton Paar MCR301 rheometer, Testometric M350-10CT textile analyzer (Rochdale, England), DLS (Malvern, England), optical microscope and scanning electron microscope (Zeiss, Germany) were used for experiments.

### 4.2. Physicochemical Characteristics of Sesame Seeds

Sesame seeds were measured for moisture, ash, protein, carbohydrate and fat. The moisture, fat, and ash content were determined according to the methods of the Association of Official Analytical Chemists (AOAC) [38], numbers 9250.10, 930.09, and 942.05, respectively. For determination of moisture content, 2 g of sesame seeds were weighed and placed into a pre-weighed dish and dried in an oven at 105 °C unit a constant weight was reached, The data reported represented by three determinations. This extraction (Soxhlet method) was carried out to estimate oil content. To determine ash content, 2 g of sample, in a crucible, was ignited and incinerated in the furnace at about 550 °C for 8 h. Total ash was expressed as a percent of dry weight. The Kjeldahl method was used to determine the crude protein of sesame seeds based on AOAC number 920.152, % (N × 6.25). The carbohydrate content of the sample was determined by difference, that is, as the difference between the total summations of percentage moisture, fat, fiber, protein, Ash and 100 [39].

### 4.3. Extraction of Sesame Seeds Protein and Determination of the Extraction Efficiency

El-Adawy [40] described a method for separating the sesame protein isolate. For this purpose, n-hexane was first used to reduce the milled seeds. Drying, grinding, and sieving of the non-fat materials were performed after disinfection. For extraction, 0.5% sodium hydroxide (*w/v*) was used at pH 11 for 1 h, after which a 20-min centrifugation at 3000 rpm was performed. Once the supernatant was separated, its pH was lowered to 4.5. The mixture was centrifuged for 15 min at 7000 rpm. The precipitates were neutralized and washed to pH 7. The sesame protein isolates were stored at 4 °C in polyethylene bags after being dried at 40 °C in a vacuum oven, ground, and sieved. Based on Equation (1), the efficiency of protein extraction from sesame seeds was determined [41].

(1) $$Protein\;Extraction\;Efficiency\;\left(\%\right)=\frac{The\;amount\;of\;protein\;in\;mixed\;protein\;solid}{The\;amount\;of\;protein\;in\;raw\;sesame\;in\;grams}\times 100$$

### 4.4. Extraction of Sesame Seeds Oleosome and Extraction Efficiency

Clean sesame seeds are mixed with 0.5 M sodium chloride solution at a ratio of 1:5 (*w/v*) and stored for 24 h. Next, sodium hydroxide (1 M) was used to raise the pH to 9. The mixture was stirred for 3 h with a magnetic stirrer at a constant speed of 1200 rpm, and then mixed for 1 min with a kitchen mixer. After filtering, the filtrate was mixed with a sodium chloride solution at 1:5 (*w/v*) ratio. The pH was elevated to 9 with 1 M hydroxide and refined again by a bilayer filter. For 20 min, the filtrate solutions were mixed and centrifuged at 3000× *g*. Centrifugation separated the mixture into two phases, liquid and solid. For protein extraction, the liquid part was separated. The creamy part was mixed with sucrose solution at a 1:2 ratio so that the final concentration was 0.5 M and centrifuged at 3000× *g* for 30 min for washing. The creamy part was then mixed with sucrose solution at 1:2 ratios, at a final concentration of 0.5 M, centrifuged for 30 min at 15,000× *g*, whose fat percentage was standardized by distilled water and then pasteurized at 98 °C for 30 min.

To determine the efficiency of sesame seed oleosome extraction, Equation (2) was used [41,42].

(2) $$\mathrm{Oleosome}\;\mathrm{Extraction}\;\mathrm{Efficiency}\left(\%\right)=100-100\times \frac{M_{\mathrm{OF}}}{M_{\mathrm{OE}}}$$

M_OF_ = grams of oil remaining in the solid, M_OE_ = grams of sesame oil of seeds

#### 4.4.1. Determination of Oleosomes Protein

Kjeldahl digestion method was applied to determine the protein content. Initially, 8 g of the powder was weighed and transferred to a 500 mL balloon for Kjeldahl digestion. Then, 8 g of Kjeldahl catalyst (per 100 g of this compound, 96 g of anhydrous sodium sulfate, 0.5 g of selenium dioxide plus 3.5 g of anhydrous sodium sulfate), and 20 mL of concentrated sulfur were added. The mixture was heated at a high temperature until a bright and clear color developed. In each balloon, 100 mL of distilled water was added once the contents were cooled. In a separate Erlenmeyer flask, 50 mL of 2% boric acid was poured. The balloons were heated whose outlets were attached to the boric acid flask and heated until green boric acid was visible. Thereafter, Erlenmeyer was separated and its contents were titrated with 0.1 N sulfuric acid. The titration was continued until the stability of the red color was achieved. Finally, the nitrogen percentage and the protein amount were calculated (Equations (3) and (4)) [38,42].

V_C_ = The volume of acid consumed for the control sample (mL)
V_S_ = The volume of acid consumed for the sample (mL)
M_S_ = Mass of sample in grams

(3) $$\mathrm{Nitrogen}\left(\%\right)=\frac{N\times 14\times 100\times \left(V_{S}-V_{C}\right)}{1000\times M_{S}}$$

(4) Protein(%)=Nitrogen(%)×5.3

#### 4.4.2. Protein Characterization by Sodium Dodecyl Sulfate-Polyacrylamide Gel Electrophoresis (SDS-PAGE)

To determine the surface membrane protein composition of oleosome droplets, the first 2 g of cream sample in a buffer (1:4) containing 50 mM tris hydrochloride, 5 M urea, 1% (*w/w*) sodium dodecyl sulfate, and 4% (*w/w*) 2-mercaptoethanol were dispersed at pH = 8. After incubation for 1 h, an equal volume of electrophoresis buffer sample containing 125 mM tris hydrochloride, 2 M urea, 1% (*w/w*) sodium dodecyl sulfate, 20% (*w/w*) glycerol, and 4% (*w/w*) 2-Mercaptoethanol were added and the mixture was heated at 98 °C for 2 min. The mixture was then frozen and thawed three times. Finally, the supernatant (mixed substrate) containing dissolved proteins was analyzed using 1-sodium dodecyl sulfate-polyacrylamide gel electrophoresis using Mini SDS Page Biorad [4]. Gel protein bands were stained with Kumasi Blue for 4 h on a shaker. The dye solution was then discarded and placed in the decolorizer solution containing methanol, acetic acid, and water for 2 h until the gel was completely discolored and protein bands appeared, whereby the protein was evaluated using a photo-transluminator.

#### 4.4.3. Measurement of Oleosome Fat 

To determine the amount of fat in the extracted oleosomes, the Soxhlet method and petroleum ether solvent were used with the amount of fat calculated using Equation (5) [43,44].

(5) $$\mathrm{Fat}\left(\%\right)=\frac{M_{O}}{M_{S}}\times 100$$

M_O_ = grams of oil extracted
M_S_ = weight of the sample in grams

#### 4.4.4. Determination of Oleosome Moisture

A certain amount of samples was poured into a container that had already reached a constant weight and weighed, with the container placed at 105 °C until a constant weight was achieved. The sample was removed from the oven and cooled in a desiccator, whereby the moisture content was calculated using Equation (6) [38,42]. m0 and m1 represent the primary and secondary mass, respectively.

(6) $$\mathrm{moisture}\left(\%\right)=\frac{m_{0}-m_{1}}{m_{0}}\times 100$$

#### 4.4.5. Measurement of Oil Droplet Size and Polydispersity Index (PDI)

Dynamic light scattering (DLS) devices were used to determine the droplet plus particle distribution and polydispersity index (PDI) of the oleosome. Under vigorous agitation with a mechanical agitator, the samples were diluted with distilled water to a fat content of 0.005% (*w/w*) before the measurements were done [4].

#### 4.4.6. Determining the Zeta Potential of Oleosomes Surface

Initially, in distilled water, 0.01 mg/mL of oleosome was dissolved and the zeta potential of the oleosome surface was measured between 2 and 12 pH levels using a Malvern model zeta-analyzer [33].

#### 4.4.7. Determination of Creaming Indexes

Creaming indices were used to evaluate the oleosome stability. To determine the creaming index, the oleosome-containing cream was carefully mixed with Britton-Robinson buffer solutions at pH ranges of 2 to 12 to a concentration of 2% (*w/w*). A 10 mL suspension of oleosomes was poured into a glass cylinder and capped after storage at room temperature (25 °C) for 7 days. For each suspension, two layers would remain visible: a layer of “cream” on the surface and a layer of “serum” at the bottom of the cylinder. The creaming index was calculated using Equation (7) [45].

(7) $$\mathrm{Creaming}\;\mathrm{index}=\frac{\mathrm{Serum}\;\mathrm{Height}}{\mathrm{Total}\;\mathrm{Height}}\times 100$$

#### 4.4.8. Oleosomes Morphology

An oleosome sample was examined under a light microscope (Zeiss model). First, 1 g of the sample was diluted in 5 mL of distilled water and was stained with Rh-DOPE (Lissamine rhodamine B sulfonyl dioleoylphosphatidylethanolamine), whereby 10 µL was placed under a microscope slide and observed. The observed particle size was calculated using Image-Pro Plus [46].

### 4.5. Preparation of Gel-Forming Samples

To prepare the composite gel samples, pure gelatin, and protein extracted from sesame seeds and sesame seed oleosome were used. For all formulas, the calculations have been based on 100 g of gel. Table 2 reports the formulation of each gel sample. According to Table 2, different ratios of sesame protein to gelatin (*w/w*) and distilled water were used. The solution was kept at room temperature for 2 h to hydrate. Then it was stirred for 1 min with a mechanical stirrer at a constant speed of 200 rpm. The final gel was prepared by mixing different weight ratios of oleosome at 50 °C [4].

#### 4.5.1. Temperature Scanning Test

During the temperature scanning test, the values of storage modulus (G′) and loss modulus (G″), which represent the mechanical properties of the material, were calculated by an oscillating test at a constant speed (constant frequency) as the temperature dropped. Accordingly, the process temperature was reduced from 50 °C to 4 °C at the constant strain (1%), constant frequency (1 Hz), and constant angular frequency (6.28 rad/s) in the linear viscoelastic region. Using a rheometer (Anton Paar MCR—301), each gel sample in this area was measured tissue indices G′, and G″ as well as complexity viscosity (η*) [4].

#### 4.5.2. Texture Analysis

The tissue analysis of the prepared gel was measured using a testometric (Instron, model M350, 10CT, England) texture analyzer at room temperature. A rod probe with a diameter of 13 mm, a movement speed of 100 mm/min and a penetration depth of 15 mm was used. The results were reported based on gel hardness, adhesiveness and breaking force. The hardness of the gel is determined by measuring the maximum height of the penetration curve of the plunger. Adhesion force is the maximum negative force to remove the plunger from the gel. Also, the breaking force, the first significant breaking in the plunger diagram inside the gel is determined at a total displacement of 15 mm [47].

#### 4.5.3. Study of Gel Structure

The microstructure of composite gels containing sesame seed oleosome was investigated using a light microscope (Zeiss Carl model, Oberkochen, Germany). The samples were stained with rhodamine at a ratio of 1 to 10 and observed by a microscope [4].

### 4.6. Statistical Analysis

All experiments were done in triplicates and the results are indicated as an average mean ± standard deviation. A significant difference between the means was defined by Duncan’s multiple comparison test using the statistical software Statistical Package for the Social Sciences Version 26 (SPSS Inc., Chicago, IL, USA).

## Figures and Tables

**Table 1:** Textural and mechanical parameters of composite gel samples.

Parameter		Breaking Force (N)	Adhesiveness (Kgf.s)	Hardness (N)
	Treatment			
S0		0.00 b ± 0.05	0.00 d ± 0.07	0.00 d ± 0.24
S1		0.01 b ± 0.05	0.01 c ± 0.08	0.01 e ± 0.22
S2		0.02 b ± 0.07	0.01 a ± 0.22	0.01 c ± 0.44
S3		0.03 b ± 0.08	0.01 a ± 0.21	0.07 b ± 0.61
S4		0.02 a ± 0.17	0.01 b ± 0.18	0.02 a ± 1.29

Different lowercase letters show a significant difference (*p* < 0.05) in the 95% probability level in each column.

**Table 2:** Mixtures of gelatin and sesame seed proteins containing oleosomes extracted from sesame seeds.

Samples	Water (g)	Gelatin (g)	Sesame Protein (g)	Sesame Protein/Gelatin	Sesame Seed Oleosome (% Based on Total Mass)
S0	96	4	-	0:100	-
S1	96	2	2	50:50	-
S2	86	2	2	50:50	10
S3	76	2	2	50:50	20
S4	66	2	2	50:50	30

## Data Availability

The data presented in this study are available on reasonable request from the corresponding author.
